# Prevalence of depression and associated risk factors among persons with type-2 diabetes mellitus without a prior psychiatric history: a cross-sectional study in clinical settings in urban Nepal

**DOI:** 10.1186/1471-244X-13-309

**Published:** 2013-11-15

**Authors:** Kiran Niraula, Brandon A Kohrt, Meerjady Sabrina Flora, Narbada Thapa, Shirin Jahan Mumu, Rahul Pathak, Babill Stray-Pedersen, Pukar Ghimire, Bhawana Regmi, Elizabeth K MacFarlane, Roshni Shrestha

**Affiliations:** 1 Department of Epidemiology and Biostatistics, Bangladesh University of Health Sciences (BUHS), Dhaka, Bangladesh; 2 Department of Neurosurgery, King Edward Medical University (KEMU)/Mayo Hospital, Lahore, Pakistan; 3 Department of Psychiatry and Behavioral Sciences, Duke University, Durham, NC, USA; 4 Duke Global Health Institute, Duke University, Durham, NC, USA; 5 Department of Epidemiology, National Institute of Preventive and Social Medicine, Dhaka, Bangladesh; 6 Department of Community Medicine, Nepalese Army Institute of Health Sciences, Kathmandu, Nepal; 7 Department of Epidemiology, Bangladesh University of Health Sciences, Dhaka, Bangladesh; 8 Institute of Medicine, Tribhuvan University Teaching Hospital, Kathmandu, Nepal; 9 Institute of Clinical Medicine, University of Oslo - Division of Women and Children, Rikshospitalet, Oslo University Hospital, Oslo, Norway; 10 Nepal Medical College, Kathmandu, Nepal; 11 Om Hospital and Research Center, Kathmandu, Nepal; 12 Kathmandu Medical College, Kathmandu, Nepal

**Keywords:** Diabetes, Depression, Glycated hemoglobin, Global mental health, Beck Depression Inventory, Developing country, South Asia, Nepal

## Abstract

**Background:**

Diabetes is a growing health problem in South Asia. Despite an increasing number of studies exploring causal pathways between diabetes and depression in high-income countries (HIC), the pathway between the two disorders has received limited attention in low and middle-income countries (LMIC). The aim of this study is to investigate the potential pathway of diabetes contributing to depression, to assess the prevalence of depression, and to evaluate the association of depression severity with diabetes severity. This study uses a clinical sample of persons living with diabetes sequelae without a prior psychiatric history in urban Nepal.

**Methods:**

A cross-sectional study was conducted among 385 persons living with type-2 diabetes attending tertiary centers in Kathmandu, Nepal. Patients with at least three months of diagnosed diabetes and no prior depression diagnosis or family history of depression were recruited randomly using serial selection from outpatient medicine and endocrine departments. Blood pressure, anthropometrics (height, weight, waist and hip circumference) and glycated hemoglobin (HbA_1c_) were measured at the time of interview. Depression was measured using the validated Nepali version of the Beck Depression Inventory (BDI-Ia).

**Results:**

The proportion of respondents with depression was 40.3%. Using multivariable analyses, a 1-unit (%) increase in HbA_1c_ was associated with a 2-point increase in BDI score. Erectile dysfunction was associated with a 5-point increase in BDI-Ia. A 10mmHg increase in blood pressure (both systolic and diastolic) was associated with a 1.4-point increase in BDI-Ia. Other associated variables included waist-hip-ratio (9-point BDI-Ia increase), at least one diabetic complication (1-point BDI-Ia increase), treatment non-adherence (1-point BDI-Ia increase), insulin use (2-point BDI-Ia increase), living in a nuclear family (2-point BDI-Ia increase), and lack of family history of diabetes (1-point BDI-Ia increase). Higher monthly income was associated with increased depression severity (3-point BDI-Ia increase per 100,000 rupees, equivalent US$1000).

**Conclusions:**

Depression is associated with indicators of more severe diabetes disease status in Nepal. The association of depression with diabetes severity and sequelae provide initial support for a causal pathway from diabetes to depression. Integration of mental health services in primary care will be important to combat development of depression among persons living with diabetes.

## Background

Diabetes mellitus is a growing public health concern in Asia, where more than 110 million people are living with diabetes, and more than 1.0 million people die annually in the region from the disorder [1]. In South Asia, greater population-level affluence is associated with an increase in health compromising behaviors related to chronic diseases such as cardiovascular disease, cancer, and diabetes [2]. With prevalence greater than 6% and growing rapidly in South Asia, the expected prevalence increase in India is 171% from 2007 to 2025 [1].

Worldwide, the prevalence of mood and anxiety disorders is higher among persons living with diabetes compared to those without diabetes [3-5]. In a meta-analysis of 42 published studies comprised of 21,351 adults, the prevalence of major depression in people with diabetes was 11% and the prevalence of clinically relevant depression was 31% ([4], c.f. [6]). The bidirectional relationship of diabetes with depression is presented by many studies [7-9]. Co-morbid depression among persons living with diabetes is associated with poor markers of diabetes control, such as glycemic control [10], retinopathy, nephropathy, neuropathy, micro-vascular complications and sexual dysfunction [11]. Depression is more prevalent among females with diabetes than males with diabetes [12], patients with type 2 versus type 1 diabetes [13] and those who are treated with insulin [14]. Depression in patients with both type 1 and type 2 diabetes is associated with psychosocial stressors of chronic medical condition [15]. An increased risk of type 2 diabetes in individuals with depression is likely due to increased counter-regulatory hormone release and action, alterations in glucose transport function, and increased immune-inflammatory activation [16]. Patients with diabetes have lower quality of life ratings, with the greatest quality reduction when depression and diabetes are comorbid [17,18].

The association of depression and diabetes has been reported in South Asia. In India, a hospital-based study suggested a prevalence of depression ranging from 8.5% to 32.5% depending upon the scales used [19]. In India, depression among persons living with diabetes has been associated with age, obesity, increased pill burden and complications of neuropathy and retinopathy [20], as well as somatic symptoms among females, and genital symptoms among males [19]. In Pakistan, history of gestational diabetes mellitus, nuclear family, obesity, marital status, history of smoking and history of high blood pressure were risk factors for depression among type 2 diabetes mellitus patients [21]. In Bangladesh, prevalence rates of depression among persons living with diabetes ranges from 28-34% [12,22,23] with differences by gender, e.g. 22% prevalence in males and 35% prevalence in females [12]. Risk factors in Bangladesh include being unmarried, insulin use, and poor glycemic control.

In Nepal, no studies to date have investigated the prevalence of depression and diabetes comorbidity. The prevalence of diabetes in Nepal is estimated to be 436,000 (2%) in 2000 and it is projected to affect 1,328,000 persons (10% prevalence) in 2030 [24]. However, in another study of a semi-urban sample, the current prevalence of type 2 diabetes mellitus in Nepal was identified as 9.5% with impaired fasting glucose having a prevalence of 19.2%. Prevalence rates were highest among middle-aged and older men [25]. Other studies in Nepal have suggested prevalence rates of 15% among persons 20–39 years old and 19% among 40 years and older [26]. In Bir Hospital, the central government hospital in Kathmandu, diabetes was reported to be the seventh most common reason for medical admissions with 2.5% prevalence [27], whereas in Tribhuvan University Teaching Hospital in Kathmandu, diabetes comprised 9.5% of total medical admissions per year [28].

The aim of this cross-sectional study was to identify the prevalence of depression among type 2 diabetes patients not previously diagnosed with a mood disorder, as well as to identify risk factors for depression among persons with diabetes. We were specifically interested in the causal pathway from diabetes to depression. Therefore exclusion criteria were designed to minimize the number of participants for whom depression may have preceded diabetes. The study focuses on clinical populations with the goal of providing prevalence estimates and risk factor associations to guide clinical prevention and management of mood disorders among persons with diabetes who do not have a psychiatric history.

## Methods

### Setting

Nepal is among the world’s poorest countries [29] with a GDP of $260 per year, which is unequally divided. Nepal recently endured the People’s War fought between the Communist Party of Nepal (Maoists) and government security forces from 1996 through 2006. Thapa and Sijapati [30] state that the economy of Nepal has favored the urban, the rural rich, and elites. Moreover, economic and political power is centralized in the capital, with minor representation from rural regions. In addition, Nepal tops the gender inequality index in South Asia, with a greater workload burden for women than men, lower literacy rates, earlier average mortality, and a myriad of discriminatory laws [31]. Ethnic inequality, institutionalized through the Hindu caste system, is represented by the lack of ethnic diversity in government positions. In 2001, 98% of people passing civil service examination were from Hindu hill groups, even though they constitute 29% of the population [30].

Community-based epidemiological studies in Nepal suggest a prevalence of depression ranging from 28% to 41% [32,33], with rates considerably greater among women, members of lower caste (Dalit) groups [34,35], the elderly and medically ill. While these high rates are influenced by the recent history of political violence, a study examining depression pre and post-war found no change in rates due to conflict related violence. This suggests that endemic poverty, lack of healthcare, and discrimination against women and persons from lower castes are chronic risk factors for poor mental health predating the conflict [33]. One study examined the association of psychosomatic complaints among persons with depression, finding that two-thirds of psychosomatic complaints were either medical comorbidities or medical conditions that cause both somatic complaints and psychiatric problems. Diabetes was a common comorbidity with depression [36].

### Participants

A cross-sectional study was conducted in three urban medical centers in Nepal’s capital city Kathmandu: (1) medical university (Tribhuvan University Teaching Hospital), (2) private medical college (Nepal Medical College) and (3) private hospital (Om Hospital and Research Center). The diversity of sites created the ability to obtain a range of participants across socioeconomic levels from 75 districts of Nepal.

### Inclusion and exclusion criteria

Every third patient with type 2 diabetes from the endocrine and medicine outpatient departments in all three centers was recruited. Patients were required to have a diagnosis of at least three months duration. Patients with chronic medical illnesses before detection of diabetes, pregnant women, and patients with a psychiatric history were excluded. Criteria for psychiatric history included history of taking anti-depressant medication, any prior diagnosis of depression, and patients with a family history of depression or mental illness. Patients with a family history of depression or other mental illness were excluded because the study examined depression as a secondary condition to diabetes. To investigate risk factors for current depression symptoms among anti-depressant naïve patients, patients currently taking anti-depressant medication were excluded because medication may reduce depression symptom burden, thus reducing the association of risk factors with current symptoms.

### Ethical approval

The study was conducted from June 2011 through June 2012 and participant recruitment was active from November 2011 through April 2012. Ethical approval was obtained from the Nepal Health Research Council and the Bangladesh Diabetes Association Ethical Review Committee. Permission was obtained from hospital representatives to allow recruitment at their respective facilities. Participants were provided with informed consent forms. If level of literacy was a concern, the consent form was read aloud to the participants.

### Instruments

In previous studies, the Beck Depression Inventory (BDI) has been used as a tool to accurately assess depressive symptoms among patients with type 2 diabetes mellitus [37,38]. The Nepali version of Beck Depression Inventory (BDI-Ia) was used for assessing depression among persons living with diabetes. The BDI-Ia was validated for use with Nepali speakers with sensitivity 0.73 and specificity of 0.91 at a cut-off score of 20 indicating clinical levels of symptoms severity [35,39,40]. Test re-test reliability was 0.90 and internal reliability was 0.92.

A semi-structured, pre-tested questionnaire was administered by the interviewers to collect information on socio-demographic characteristics, disease variables, and behavioral characteristics. The questionnaire was prepared in English and translated into Nepali language and was reviewed by Nepali psychiatrists, endocrinologists, epidemiologists, and biostatisticians. Pre-testing of the questionnaire was performed to gather information about understandability, time consumed by each question, consistency among related variables and acceptability. After reviewing the outcome of pre-testing, changes were incorporated accordingly. Medical interns and nurses in the outpatient departments were given a short course on data collection using the instruments, including anthropometric measurements (BMI, WHR), clinical parameters (blood pressure), and use of the Nepali BDI-Ia. Height, weight, waist circumference and hip circumference were measured using standard clinical protocols.

After the interviews, patients were then taken to the hospital laboratory for blood draws for analysis of HbA_1c_ using 2 ml of venous blood. The local supervisor, a professor of epidemiology, reviewed all procedures and notation at the end of each day.

### Statistical analyses

Sample size was estimated using the prevalence of depression among persons with type 2 diabetes from a recent study conducted in Bangladesh at a tertiary care center [22]. Using an estimated prevalence of 35%, 95% confidence intervals, and absolute precision of 5%, a sample size of 348 was determined. This study recruited 385 patients.

Statistical comparisons of socio-demographic variables between male and female patients were conducted using chi-squared tests and t-tests for categorical and continuous variables respectively. Odds ratios (OR) with 95% confidence interval (CI) were calculated for categorical comparisons. All tests were two-tailed and with *p* < 0.05 considered statistically significant. Bivariate and multivariable statistical analyses were conducted using linear regression. Regression coefficients (β) and 95% CI are presented for multivariable and bivariate linear regressions with total BDI-Ia score as the dependent outcome. Multiple linear regressions were performed to adjust for potential confounding factors.

The final multivariable model included sex, age, religion, ethnicity/caste, marital status, years of education, monthly income, family type, number of family members, parental history of DM, family history of HTN, personal history of HTN, smoking, alcohol use, treatment non-adherence, DM complications, erectile dysfunction, systolic blood pressure, diastolic blood pressure, waist-hip-ratio, and glycated hemoglobin. These variables were included in the model because of a known history of association with depression in Nepal or other study populations. Body-mass-index was not included in the multivariable model because it was not statistically significant in bivariate analysis with depression and because of high collinearity with waist-hip-ratio. Although systolic and diastolic blood pressures are collinear, when entered in the same multivariable model, they both remained significant. Therefore, both were kept in the model.

## Results

### Sample characteristics

The sample included 48.3% men and female 51.7% women (see Tables 1 and 2). The mean *±* standard deviation (SD) for age was 52.0 ± 13.1 years, with males 6 months older than females, which was not a statistically significant difference (t = 0.4, df = 383, p = 0.7). The majority of participants practiced Hinduism (76.6%) and 17.4% practiced Buddhism. Nearly half (45%) of the participants belonged to high caste groups (Bahun, Chhetri), more than two-thirds of participants were married (68.6%), and the majority lived in nuclear families (79.2%). Among total participants, 47.5% had a family size less than five members. Mean total years of formal education was 5.8 ± 6.7, with only 52% of participants having any formal education and only 20% with high school graduate equivalent degrees. Illiteracy was greater among female (25.6%) than males (18.1%). Median monthly income of participants was Nepali Rupiyaa 10,000, range 0–200,000, (NRs. 100 = US$1). Median family income was Rs. 30,000, range Rs. 0 – 500,000.

**Table 1:** Categorical demographics

**Variables**	**Total**	**Male**	**Female**	**Sex difference**	**P value**
	**No. (%)**	**No. (%)**	**No. (%)**	**OR (95% CI)**	
Sex					
Male	186 (48.3)	-	-	-	-
Female	199 (51.7)	-	-		
Religion					
All others	90 (23.4)	50 (13.0)	40 (10.4)	Ref.	
Hindu	295 (76.6)	136 (35.3)	159 (41.3)	1.46 (0.91,2.35)	0.12
Ethnicity					
High caste	173 (45.0)	83 (21.60)	90 (23.4)	Ref.	
Newar	65 (16.9)	27 (7.0)	38 (9.9)	1.29 (0.73,2.31)	0.38
Hilly indigenous (Janajati)	90 (23.4)	54 (14.0)	36 (9.4)	0.62 (0.37,1.03)	0.07
Occupational castes (Dalit)	11 (2.9)	8 (2.1)	3 (0.8)	0.35 (0.09,1.35)	0.13
All others	46 (11.9)	14 (3.6)	32 (8.3)	2.11 (1.05,4.23)	0.04
Marital status					
Married	264 (68.6)	138 (35.8)	126 (32.7)	Ref.	
Others	121 (31.4)	48 (12.5)	73 (19.0)	1.66 (1.07,2.57)	0.02
Residence					
Rural	60 (15.6)	22 (5.7)	38 (9.9)	Ref.	
Urban	242 (62.9)	108 (28.1)	134 (34.8)	0.72 (0.40,1.29)	0.27
Sub-urban	82 (21.3)	56 (14.5)	26 (6.8)	0.27 (0.13,0.54)	<0.001
Occupation*					
Unemployed	8 (2.1)	2 (0.5)	6 (1.56)	Ref.	
House wife	125 (32.5)	60 (15.6)	65 (16.9)	0.36 (0.07,1.86)	0.22
Farmer	59 (15.3)	28 (7.3)	31 (8.1)	0.37 (0.07,1.98)	0.25
Service	32 (8.3)	15 (3.9)	17 (4.4)	0.38 (0.07,2.16)	0.27
Retired	86 (22.3)	46 (11.9)	40 (10.4)	0.29 (0.01,1.52)	0.14
Laborers	14 (3.6)	8 (2.1)	6 (1.6)	0.25 (0.04,1.70)	0.16
Business	44 (11.4)	20 (5.2)	24 (6.2)	0.40 (0.07,2.20)	0.29
All others	14 (3.6)	7 (1.9)	7 (1.8)	0.33 (0.05,2.26)	0.26
Education					
No formal	182 (47.3)	85 (22.07)	97 (25.20)	Ref.	
Below primary	38 (9.9)	15 (3.9)	23 (6.0)	1.34 (0.66,2.74)	0.42
Primary/secondary	57 (14.8)	27 (7.0)	30 (7.8)	0.974 (0.54,1.77)	0.93
School leaving certificate	108 (28.1)	59 (15.3)	49 (12.7)	0.73 (0.45,1.18)	0.19
Family type					
Nuclear	305 (79.2)	148 (38.4)	157 (40.8)	Ref.	
Joint	80 (20.8)	38 (9.9)	42 (10.9)	1.04 (0.64,1.71)	0.87

*Two individuals were students. Results did not include these two individuals in occupation analysis because of small sample and unstable confidence interval.

**Table 2:** Continuous demographics

**Continuous variables**	**Range**	**Mean ± SD**			**Sex difference (*T*-test value)**	**95% Confidence interval**	***p*** **value**
		**Total**	**Male**	**Female**			
Age (years)	23-100	52.0 ± 13.1	52.2 ± 12.6	51.7 ± 13.5	0.40	-2.10, 3.15	0.69
Education (years)	0-19	5.8 ± 6.7	6.3 ± 6.9	5.4 ± 6.5	1.29	-0.46, 2.22	0.20
No of members in family	1-20	5.0 ± 3.0	5.1 ± 3.1	4.8 ± 2.8	1.21	-0.23, 0.96	0.23
Monthly income (thousand rupees)	0-200	17.9 ± 37.0	18.7 ± 39.9	17.2 ±34.2	0.40	-59.20, 89.39	0.69
Monthly family income (thousand rupees)	0-500	50.7 ± 68.2	43.9 ±5.6	57.0 ± 77.3	-1.89	-26.73, 51.69	0.06
Monthly family expenditure (thousand rupees)	0-130	28.7 ± 25.9	26.7 ± 24.1	30.6 ± 27.3	-1.51	-91.47, 12.09	0.13
Monthly diabetes related health care cost (thousand rupees)	0-35	2.2 ± 4.3	2.1 ± 4.2	2.3 ± 4.5	-0.48	-1.08, 0.66	0.63

Note: 1000 Nepali Rupees is equivalent to approximately 10 US dollars.

### Depression

The mean and standard deviation for total BDI-Ia score of participants was 19.8 ± 8.0. Male and female scores were 18.6 ± 7.6 and 20.9 ± 8.1 respectively. Women had significantly greater BDI-Ia scores compared to men (t = -2.74, df = 383, p < 0.01). The number of participants with clinical depression (≥20 BDI-Ia score) was 155 (40.3% of the total sample). Among depressed patients, 48.2% were female and 31.7% were male. BDI-Ia scores above the cut-off were significantly associated with sex (*χ*^2^ = 10.91, df = 383, p < 0.001).

In bivariate analyses, socio-demographic, economic, anthropometric, clinical and biochemical parameters were significantly associated with BDI-Ia score (See Tables 3 and 4). Female sex associated with a β = 2.2 point in total BDI-Ia score compared to men (df = 383, p = 0.006). Participants having family history of diabetes showed a protective effect of β = -2.7 (95% CI: -4.3,-1.0, df = 383, p < 0.01) compared to persons without family history of diabetes (p = 0.02). Patients with at least one diabetic complication had a 3.6 point higher BDI-Ia score (95% CI: 2.0, 5.2, df = 383, p < 0.001) and men with erectile dysfunction had greater BDI-Ia scores (β: 8.9; 95% CI: 4.4, 13.4, df = 185, p < 0.001). As the duration of diagnosis of diabetes increased BDI-Ia score decreased (β: -0.1; 95% CI: -0.3,-0.01, df = 383, p = 0.03), i.e. persons who were further along in the disease course, patients reported fewer depression symptoms. Other significant characteristics were systolic BP (β: 0.3; 95% CI: 0.3, 0.4, df = 383, p < 0.001), diastolic BP (β: 0.4; 95% CI: 0.4, 0.5, df = 383, p < 0.001), and WHR (β: 18.5; 95% CI: 7.7, 29.4, df = 383, p < 0.001). BMI (β: -0.01; 95% CI: -0.3,-0.01, df = 383, p = 0.90) was not significant in bivariate comparisons.

**Table 3:** Bivariate regression of categorical variables with depression symptom severity (total BDI-Ia score)

**Characteristics**		**Total samples**		
**Variables**	**Categories**	**Mean (SD)**	**Beta (95% CI)**	**P Value**
Sex	Male	18.6 ± 7.6	Ref.	<0.01
	Female	20.9 ± 8.1	2.2 (0.6, 3.8)	
Religion	All others	21.3 ± 8.7	Ref.	0.03
	Hindu	19.3 ± 7.7	-2.1 (-4.0,-0.2)	
Ethnicity	High caste	19.3 ± 7.3	Ref.	
	Newar	19.6 ± 8.4	-0.7 (-3.3,1.9)	0.62
	Hilly indigenous (Janajati)	20.5 ± 8.6	-0.4(-3.4,2.6)	0.80
	Occupational castes (Dalit)	21.1 ± 10.7	0.5 (-2.4,3.3)	0.74
	Others	20.0 ± 7.7	1.1 (-4.2,6.4)	0.68
Marital status	Married	20.6 ± 8.2	Ref.	
	All others	19.41 ± 7.9	-1.2 (-2.9,0.5)	0.17
Residence	Rural	20.2 ± 7.8	Ref.	
	Urban	20.3 ± 8.3	2.3 (-0.4,4.9)	0.09
	Sub-urban	17.9 ± 6.7	2.4 (0.4,4.5)	0.02
Occupation	Unemployed	20.4 ± 17.9	Ref.	
	House wife	19.6 ± 8.0	2.9 (-4.1,9.8)	0.42
	Farmer	21.7 ± 6.7	2.1 (-2.4,6.5)	0.36
	Service	19.1 ± 9.1	4.2 (-0.5,8.9)	0.08
	Retired	19.3 ± 7.3	1.6 (-3.4,6.6)	0.53
	Student	20.0 ± 5.7	1.8 (-2.8,6.3)	0.44
	Laborers	18.5 ± 10.0	2.5 (-9.4,14.4)	0.68
	Business	20.2 ± 7.0	1.0 (-4.9,6.9)	0.74
	All others	17.5 ± 6.5	2.7 (-2.1,7.6)	0.27
Education	No formal	20.7 ± 7.7	Ref.	
	Formal but below primary	20.2 ± 7.1	2.7 (0.8,4.6)	<0.01
	Primary/secondary	20.2 ± 7.7	2.3 (-0.6,5.2)	0.12
	SCL + (class 10 passed)	17.9 ± 8.6	2.2 (-0.3,4.8)	0.09
Family type	Nuclear	19.8 ± 7.5	Ref.	
	Joint	19.9 ± 9.6	0.1 (-1.9,2.1)	0.91
Parental history of DM	No	20.7 ± 7.9	ref	
	Yes	18.0 ± 7.8	-2.7 (-4.3,-1.0)	<0.01
Treatment modality	Oral	19.5 ± 7.7	Ref.	
	Any insulin	21.5 ± 8.4	2.8 (0.5,5.2)	0.02
	All others	16.7 ± 6.9	4.77 (2.3,7.3)	<0.001
Non adherence	No	18.2 ± 7.2	Ref.	
	Yes	22.6 ± 8.5	4.4 (2.8, 6.0)	<0.001
Frequency of non adherence	≤once a week	19.8 ± 8.9	Ref.	
	>one a week	24.8 ± 7.5	5.0 (2.3,7.8)	<0.001
Any diabetic complication	No	17.9 ± 7.0	Ref.	
	yes	21.4 ± 8.4	3.6 (2.0,5.2)	<0.001
Erectile dysfunction	No	19.5 ± 7.9	Ref.	
	Yes	28.4 ± 4.8	8.9 (4.4,13.4)	<0.001
Smoking	Never	19.6 ± 7.3	Ref.	
	Past	19.0 ± 8.5	-1.2 (-3.2,0.7)	0.22
	Current	20.9 ± 8.6	-1.7 (-4.1,0.3)	0.10
Alcohol use	Never	19.6 ± 7.7	Ref.	
	Past	20.3 ± 7.5	-0.1 (-2.1,1.9)	0.93
	Current	19.7 ± 8.9	0.6 (-1.7,2.8)	0.61
History HTN	No	18.8 ± 7.68	Ref.	
	Yes	20.8 ± 8.15	2.0 (0.4,3.6)	0.01
HTN medication	No	16.2 ± 5.4	ref	
	Yes	20.9 ± 8.1	4.7 (1.0,8.5)	0.01
Family history of HTN	No	20.6 ± 8.2	Ref.	
	Yes	18.3 ± 7.8	-1.8 (-3.6,-0.1)	0.03

*Abbreviations:* BDI, Beck Depression Inventory; SLC+, School Leaving Certificate (high school graduation equivalent); HTN, hypertension; DM, diabetes mellitus.

**Table 4:** Bivariate regression of continuous variables with depression symptom severity (total BDI Score)

**Characteristics**	**Beta (95% CI)**	**p-value**
Age (years)	-0.09 (-0.15, -0.03)	<0.01
Education (years)	-0.17 (-0.29, -0.05)	<0.01
No of members in family	-0.02 (-0.29, 0.25)	0.90
Monthly income (thousand rupees)	0.01 (-0.01, 0.03)	0.33
Monthly family income (thousand rupees)	-0.01 (-0.00, 0.01)	0.85
Monthly family expenditure (thousand rupees)	-0.01 (-0.04, 0.02)	0.54
Monthly diabetes related health care cost (thousand rupees)	-0.01 (-0.00,0.01)	0.87
Duration of diagnosis of DM	-0.13 (-0.25,-0.01)	0.03
Duration of diagnosis of HTN	0.09 (-0.06,0.24)	0.26
Systolic BP	0.31 (0.27,0.35)	<0.001
Diastolic BP	0.42 (0.37,0.48)	<0.001
BMI	-0.01 (-0.21,0.18)	0.90
WHR	18.5 (7.65,29.44)	<0.001
HbA1c	3.16 (2.82, 3.49)	<0.001

*Abbreviations:* BDI, Beck Depression Inventory; HTN, hypertension; DM, diabetes mellitus; BP, blood pressure; BMI, body mass index; WHR, waist-hip ratio; HbA1c, glycated hemoglobin.

Note: 1000 Nepali Rupees is equivalent to approximately 10 US dollars.

In the multivariable regression, clinical and biochemical parameters as well as anthropometrics were significantly associated with depression symptom severity. An increase of 1 unit (%) HbA1c was associated with a BDI-Ia increase of 2.08 (See Table 5). An increase of 10 mmHg in systolic and diastolic blood pressure was associated with an increase of 1.3 and 1.4 of BDI-Ia scores, respectively. An increase of 0.1 in waist-hip-ratio was associated with a 1 point increase in BDI-Ia score. Any diabetic complication was associated with a BDI-Ia score increase of 1.2, whereas erectile dysfunction was associated with a 4.74 BDI-Ia score increase. Regarding treatment parameters, insulin use compared to oral or other treatments was associated with a 1.74 BDI-Ia score increase, and adherence was associated with a 1.05 BDI-Ia score decrease. Affluence was associated with greater depression severity. An increase of NRs. 100,000 (US$1,000) per month in personal income predicted an increase of 3 points on total BDI-Ia score. Other significant parameters included known family history of hypertension (β = -1.5, df = 362, p = .01) and living in a nuclear family (β = 1.6, df = 362, p = .03) as opposed to joint-extended family. Given the greater depression severity among persons early in the diagnosis and treatment process, a sensitivity analysis of diagnoses within the past 3 years was conducted. However, no additional risk factors were identified.

**Table 5:** Multivariable Regression for Depression Symptom Severity (Total BDI-Ia Score)

**Variables**		**Predictors for total BDI score**		
		**Beta**	**(95% CI)**	**P Value**
Intercept		-29.92	-38.81, -21.03	<0.001
Sex (female)		0.67	-0.34, 1.67	0.34
Age (years)		-0.01	-0.05, 0.03	0.72
Religion (Hindu)		-0.81	-2.21, 0.59	0.26
Ethnicity	High Caste Hindu	Ref.		
	Newar	-0.86	-2.23, 0.50	0.21
	Hill indigenous (Janajati)	-1.21	-2.75, 0.33	0.12
	Occupational castes (Dalit)	-0.40	-3.34, 2.54	0.79
	All others	0.93	-0.72, 2.58	0.27
Marital status (married)		0.69	-0.45, 1.84	0.24
Years of education		-0.06	-0.13, 0.02	0.14
Monthly income (thousand rupees)		0.03	0.01, 0.04	<0.001
Family type (nuclear)		1.60	0.21, 3.00	0.03
Total family members		0.20	-0.00, 0.38	0.05
No known parental history of DM		1.48	0.33, 2.62	0.01
Family history of HTN		-0.75	-1.89, 0.38	0.19
History of HTN		-0.31	-1.34, 0.72	0.55
Smoking	Never	Ref.		
	Past	-1.05	-2.40, 0.29	0.12
	Current	0.08	-1.32, 1.48	0.91
Alcohol use	Never	Ref.		
	Past	-0.22	-1.58, 1.14	0.75
	Current	-0.63	-2.07, 0.81	0.39
Treatment modality for DM	Oral	Ref.		
	All insulin	1.74	0.60, 2.88	<0.001
	All other	-0.81	-2.32, 0.68	0.29
Non-adherence		1.05	0.02, 2.08	0.05
DM complication		1.19	0.18, 2.21	0.02
Erectile dysfunction		4.74	1.94, 7.53	<0.01
BP Systolic (mmHg)		0.13	0.08, 0.17	<0.001
BP Diastolic (mmHg)		0.14	0.08, 0.20	<0.001
WHR		9.12	2.47, 15.77	0.01
HbA1c (%)		2.08	1.76, 2.41	<0.001

R^2^ = .4, F = 15, df = 369, p < 0.001.

*Abbreviations:* BDI, Beck Depression Inventory; HTN, hypertension; DM, diabetes mellitus; BP, blood pressure; mmHg, millimeters of mercury; BMI, body mass index; Kg/m^2^, kilograms per meter squared; WHR, waist-hip ratio; HbA1c, glycated hemoglobin.

Note: 1000 Nepali Rupee is equivalent to approximately 10 US dollars.

## Discussion

This was a cross-sectional study conducted in three clinical settings of urban Nepal. The aim of the study was to find the prevalence of depression among persons living with diabetes and to identify probable risk factors. Patients who were diagnosed with diabetes at least three months prior to the study were included. Patients with other chronic medical illnesses before detection of diabetes, pregnant women with diabetes, people having psychiatric problems before diagnosis of diabetes, people who were taking anti-depressants and people who had a family history of depression were excluded**.** The goal of these criteria was to minimize the likelihood of depression as a pre-existing condition and instead identity depression that occurred secondary to diabetes.

In the present study, the proportion of participants with BDI-Ia scores above the clinically validated cutoff in Nepal (BDI ≥ 20) was 40.3%. This rate is comparable to prevalence of depression (36.6%), as reported in a worldwide meta-analysis of studies among persons with diabetes in clinical settings [41]. The rate is higher than that observed in the United States, where prevalence of depression among persons with diabetes ranges from 2%-28% [14.] Our observed rate is comparable to Mexican-born Americans in the Texas who display a 40% prevalence rate [42] and comparable to a German study with a depression prevalence among persons with diabetes of 41.9% also using the BDI [43]. The observed rate in this study was at the upper range of other studies conducted in South Asia. Hospital-based studies from India showed prevalence ranging from 8.5%-32.5% [19]. Asghar *et al.* did a community based study in Bangladesh, which showed 27.9% depression among type 2 diabetes patients [12]. A tertiary hospital based study conducted in Bangladesh showed 34.8% depression in persons living with diabetes [22].

### Risk indicators for depression in patients with type 2 diabetes

As expected, complications of diabetes were significantly associated with depression. This has been observed across most studies of depression and diabetes in both high [44,45] and low-income settings including South Asia [21,22]. Erectile dysfunction was one of the strongest predictors of depression, which is consistent with De Groot *et al.*[11] and Lustman *et al.,*[10]. In this study family history of diabetes was associated with lower depressive symptoms than those who had no family history. While the study does not have quantitative or qualitative data to illuminate the association, it is speculated that family history may reduce fear and anxiety related to the disorder as well as normalize the experience, resulting in lower psychological distress. As with other studies, insulin use predicted greater depression, with insulin likely a proxy for greater disease severity [4,14,22,41,46-49]. The study showed non-adherence to at least one aspect of the diabetes management plan (life style, diet, medication) was strongly associated (p < 0.001) with depression, consistent with studies in high-income countries [3,17,50,51]. Studies showed not checking blood sugar [3], lack of regular exercise [45], lack of medical vigilance, and lack of dietary modification [49] were associated with depression. Time since diabetes diagnosis was inversely associated with depression severity, suggesting that the early experience of the disease is the period of greatest psychological distress and adjustments in lifestyle. High blood pressure, either systolic or diastolic, was associated with greater depression severity, similar to other findings [21,22]. Glycemic status (glycated hemoglobin) was consistently associated with depression in almost all studies done so far in research related to depression among type 2 diabetes [10,22,47,52-55]. HbA1c was strongly associated with depression in this study as well, which is also a likely proxy for disease severity.

A number of risk factors associated with depression in Nepal were not significantly associated with depression among this clinical population with diabetes. For example, gender was not a significant predictor of depression among persons with diabetes in the multivariable regression model. This contrasts with community studies of depression that show a consistently greater prevalence among Nepalese women compared to men [32,33]. In a study of women with diabetes in India, Weaver and Hadley [56] found that not fulfilling gender-specific social roles predicted greater levels of depression. In this sample, there may be no gender differences between men and women because diabetes impairs gender-role performance for both sexes, as suggested by greater depression among men living with diabetes with erectile dysfunction. Diabetes may have comparable functional consequences for both genders, whereas women in Nepal are more vulnerable socially even in the absence of physical disease [34].

Age is a strong predictor of depression in the general community in Nepal [32,33,35]. However, it was not a significant predictor in this Nepali clinical population with diabetes. Age has shown an inconsistent relationship with depression and in people with diabetes with depression in a number of studies: Goldney *et al.* also did not find an association of age with depression among persons living with diabetes [17]. Ravel *et al*. did found depression to be strongly associated with age above 54 years [20].

In this study, caste and ethnicity were not significantly associated with depression. However, community-based samples in rural Nepal show a greater preponderance of depression among low-caste Dalit groups [3,35,57-59]. In other countries, ethnic minorities with diabetes display more depression than majority groups [44,48,60,61]. Marital status also was not significant, despite being a predictor in rural Nepal and in studies of persons living with diabetes in other countries (Pakistan) [21]. This study showed participants from urban area were more depressed (p < 0.001) which is not consistent with other studies where rural population was more depressed [20,51]. It is important to note that the prevalence of depression in this sample of persons living with diabetes (40%) is comparable to rates of depression observed among other risk groups in Nepal such as low caste Dalit groups (BDI-Ia-based depression prevalence, 50%) [35], populations with high political violence exposure including both adult civilians (Dang district, BDI-Ia-based depression prevalence, 43%) [32] and child soldiers (depression prevalence, 53%) [59], and populations with high rates of structural violence including poverty, lack of education, lack of health care, and high rates of gender discrimination (Jumla district, BDI-based depression prevalence, 41%) [33].

In this study, greater personal income was associated with greater depression. Although this study showed participants with high income were more depressed (p < 0.01) than their lower income counterparts, Bell *et al.*[50], Egede *et al.*[60], Everson *et al.*[61] suggests patients with lower income have more depression. In India, mixed methods research by Mendenhall and colleagues [62] suggests that depression is greater among low-income persons with diabetes, likely influenced by both greater financial stressors and also by impaired access to diabetes care. In urban Nepal, wealth may be a proxy for poorer health overall. Whereas obesity, metabolic syndrome, and diabetes are greater risks among poor populations in high income countries, affluence is associated with these poor health outcomes in some studies in South Asia [2].

However, the rapid economic transitions in India are demonstrating that high-income groups are able to mobilize behavioral and medical resources while a growing burden of chronic health problems falls upon the middle and lower class, which is the pattern observed in high-income countries [63,64]. Therefore, wealth may be a proxy for poor health habits, poor nutrition, limited exercise, excessive caloric intake, and smoking and drinking [65] at a specific moment in time in Nepal as the burden shifts to other groups. Alternatively, the selection criteria may have confounded the association of wealth and depression, as discussed in the limitations section below.

The present study did not reveal any association between behavioral characteristics of tobacco, alcohol, and other drug use. However, this may be because personal income was a stronger proxy for a constellation of poor behaviors in this population. Most other studies have shown an association of these behaviors with depression among persons living with diabetes [17,21]. One reason that smoking may not be associated with poorer outcomes is the epidemic of respiratory disorders in Kathmandu given the overwhelming burden of pollution in the city. The present study showed an association between waist hip ratio and depression (p = 0.001), which is consistent with findings from Lustman *et al.*[10] and Larijani *et al.*[43].

### Limitations

This study has a number of limitations. The main limitation is the generalizability of findings based on the sample. The goal was to identify persons with least likelihood of pre-diabetes depression. While this is helpful to consider the pathway from diabetes to depression and useful to demonstrate the high rate of mental health problems in persons with no prior history, it does confound some of the risk factor associations reported. The association between higher economic status and depression may be the result of excluding persons with prior depression, who are more likely to be low socioeconomic status given the high rates of depression in this population. Moreover, this study does not provide new information on the pathway from depression to diabetes. Further information on this group is needed to explore how mental health treatment can interrupt the transition from psychiatric to endocrine morbidity. Ultimately, studies that include comparisons of community residing persons with and without diabetes and depression rates and risk factors is needed to determine pathways and points of intervention. The strong association of sociobehavioral risk factors with depression among this group also highlights the need for models looking at direct and indirect effects of social marginalization with multiple mediation through physical health morbidity.

## Conclusion

Depression is associated with indicators of more severe diabetes disease status in Nepal. The associations of depression with diabetic severity and sequel provide initial support for a causal pathway from diabetes to depression in Nepal. Integration of mental health services in primary care will be an important factor in preventing the development of depression among diabetes patients. Concealed depression among type-2 diabetes patients needs to be assessed in every diabetes care setting. Stepped and collaborative care models for treatment [66,67] of diabetes and depression should be explored for treatment options in Nepal and other low-income countries [23]. Moreover, education about the connection between physical health and mental health, which has been used in high-income settings [68], should be considered for psycho-education programs within this population. Cognitive Behavior Therapy (CBT) with a focus on the connection between mind and body has been adapted for use with Nepali populations [69] and should be considered for studies with patients suffering from comorbid depression and diabetes in Nepal. With lifestyle changes and increasing affluence, the risk of diabetes will likely increase on a population level. More research is needed to determine public health measures to combat greater non-communicable health risks in Nepal. Mental health, obesity, and metabolic disorders have common risk factors therefore common solutions should be pursued rather than separate approaches to prevention of mental and physical health problems [70].

## Competing interests

The authors declare no financial or non-financial competing interests.

## Authors’ contributions

KN designed the study, performed data collection, data analysis and drafted the manuscript. BK performed data analysis and drafted the manuscript. MSF contributed in conception, design and interpretation of data. NT revised the article critically and gave valuable suggestions. SJM helped in data analysis, literature search, and interpretation of data. RP contributed in acquisition of data and helped in analysis. BP helped in conception and design. PG, BR, RS contributed in acquisition of data, drafting articles and reviewed the article before submission to publishers. KN, BK, and EM significantly revised the manuscript. All authors read and approved the final manuscript.

## Pre-publication history

The pre-publication history for this paper can be accessed here:

http://www.biomedcentral.com/1471-244X/13/309/prepub
